# *Giardia* Colonizes and Encysts in High-Density Foci in the Murine Small Intestine

**DOI:** 10.1128/mSphere.00343-16

**Published:** 2017-06-21

**Authors:** N. R. Barash, C. Nosala, J. K. Pham, S. G. McInally, S. Gourguechon, B. McCarthy-Sinclair, S. C. Dawson

**Affiliations:** a Department of Microbiology and Molecular Genetics, UC Davis, Davis, California, USA; b Department of Molecular and Cell Biology, UC Berkeley, Berkeley, California, USA; University at Buffalo

**Keywords:** *Giardia*, bioluminescence, encystation, parasite, pathogenesis

## Abstract

*Giardia lamblia* is a highly prevalent yet understudied protistan parasite causing significant diarrheal disease worldwide. Hosts ingest *Giardia* cysts from contaminated sources. In the gastrointestinal tract, cysts excyst to become motile trophozoites, colonizing and attaching to the gut epithelium. Trophozoites later differentiate into infectious cysts that are excreted and contaminate the environment. Due to the limited accessibility of the gut, the temporospatial dynamics of giardiasis in the host are largely inferred from laboratory culture and thus may not mirror *Giardia* physiology in the host. Here, we have developed bioluminescent imaging (BLI) to directly interrogate and quantify the *in vivo* temporospatial dynamics of *Giardia* infection, thereby providing an improved murine model to evaluate anti-*Giardia* drugs. Using BLI, we determined that parasites primarily colonize the proximal small intestine nonuniformly in high-density foci. By imaging encystation-specific bioreporters, we show that encystation initiates shortly after inoculation and continues throughout the duration of infection. Encystation also initiates in high-density foci in the proximal small intestine, and high density contributes to the initiation of encystation in laboratory culture. We suggest that these high-density *in vivo* foci of colonizing and encysting *Giardia* likely result in localized disruption to the epithelium. This more accurate visualization of giardiasis redefines the dynamics of the *in vivo Giardia* life cycle, paving the way for future mechanistic studies of density-dependent parasitic processes in the host.

**IMPORTANCE** *Giardia* is a single-celled parasite causing significant diarrheal disease in several hundred million people worldwide. Due to limited access to the site of infection in the gastrointestinal tract, our understanding of the dynamics of *Giardia* infections in the host has remained limited and largely inferred from laboratory culture. To better understand *Giardia* physiology and colonization in the host, we developed imaging methods to quantify *Giardia* expressing bioluminescent physiological reporters in two relevant animal models. We discovered that parasites primarily colonize and encyst in the proximal small intestine in discrete, high-density foci. We also show that high parasite density contributes to encystation initiation.

## Abstract (precis)

*Giardia* is a single-celled parasite causing significant diarrheal disease in several hundred million people worldwide. Due to limited access to the site of infection in the gastrointestinal tract, our understanding of the dynamics of *Giardia* infections in the host has remained limited and largely inferred from laboratory culture. To better understand *Giardia* physiology and colonization in the host, we developed imaging methods to quantify *Giardia* expressing bioluminescent physiological reporters in two relevant animal models. We discovered that parasites primarily colonize and encyst in the proximal small intestine in discrete, high-density foci. We also show that high parasite density contributes to encystation initiation.

## INTRODUCTION

*Giardia lamblia* is a unicellular protistan parasite causing acute and chronic diarrheal disease in over 200 million people worldwide, primarily in developing countries with inadequate sanitation and water treatment (1). Giardiasis is a serious disease of children, who may experience substantial morbidity, including diarrhea, malnutrition, wasting, and developmental delay (2–4). In the United States, giardiasis is the most frequently diagnosed waterborne diarrheal disease and commonly affects travelers and immunosuppressed individuals (5). Trophozoites are not invasive, and *Giardia* infection does not produce a florid inflammatory response; however, giardiasis is associated with villus shortening, enterocyte apoptosis, hypermobility, and intestinal barrier dysfunction (6). The estimated failure rates of up to 20% for standard drug treatments such as metronidazole (7) and growing evidence of drug resistance in *Giardia* (8–10) underscore the need for new therapeutic treatments of this widespread and neglected diarrheal disease.

Motile* Giardia* trophozoites colonize and proliferate in the small intestine (SI) (11), attaching to the intestinal villi to resist peristalsis using a complex microtubule structure termed the ventral disc (12, 13). In the gut, trophozoites differentiate into infectious cysts that are eventually excreted and can contaminate water sources in the environment (5, 14). Disseminated cysts are ingested and excyst into trophozoites after passage through the stomach, completing their life cycle in the host gastrointestinal (GI) tract. Trophozoites are proposed to colonize the acidic, cholesterol-rich duodenum or jejunum and then initiate encystation when peristalsis sweeps them to the alkaline, cholesterol-depleted distal intestine (15–17). Encystation is thus believed to be triggered via cues that are specific to particular anatomical sites in the gastrointestinal tract (16, 18) and can be induced *in vitro* by increasing pH and decreasing cholesterol or by increasing bile and lactic acid in the medium (16, 19, 20). However, cysts produced *in vitro* excyst less efficiently using *in vitro* excystation protocols (21) and are less robust at establishing infections in animal models than cysts harvested directly from feces. This implies that additional host factors are required for infectious cyst production (16). As differentiation of the trophozoite into the infectious cyst is a critical aspect of *Giardia*’s pathogenesis (22), determining the extent of *in vivo* parasite differentiation to cysts and subsequent cyst dissemination is key to understanding *in vivo* host-parasite interactions (23–25).

Despite decades of study, the host-parasite infection dynamics underlying the extent and progression of acute and chronic giardiasis (4, 26–28) are poorly understood. Due to the limited accessibility of the gastrointestinal tract (15–17), our knowledge of *Giardia*’s physiology and differentiation *in vivo* is largely inferred from laboratory culture rather than *in vivo* models of the disease (15, 17). While *in vitro* studies have established that the initiation of encystation is transcriptionally controlled (8–10), understanding the complex temporospatial dynamics of the parasite life cycle and interactions with the host remains challenging. *In vitro* models of giardiasis are not necessarily adequate proxies for infection within the host as they may not accurately mirror *in vivo* parasite physiology. Further, *in vitro* studies rarely have been confirmed through analogous *in vivo* studies of parasite physiology. Thus, *in vivo* models are necessary to understand parasite infection dynamics in the host and to evaluate new antigiardial drugs.

Zoonotic* Giardia* strains have varied physiologies and have been classified into assemblages (roughly equivalent to species), including the human isolates from assemblages A (e.g., strains WBC6 and DH) and B (e.g., strains GS and H3) (29, 30). Genomes are available for the assemblage A strains WBC6 (31) and DH (32), the assemblage B strain GS (32, 33), and some human clinical isolates (34); however, reasonably robust molecular genetic tools have been developed only for *Giardia* strains WBC6 and GS (35). Animal models of giardiasis include adult (36, 37) or suckling (38) mice or adult gerbils (39) infected with either human *Giardia lamblia* isolates from assemblage A (strain WBC6) or assemblage B (strain GS or H3) or murine *Giardia muris* isolates (40). Infections with cysts are possible using commercially available assemblage B strain H3 cysts passaged through gerbils (3); however, strain H3 currently has no genome sequence and has not been demonstrated to be genetically manipulable. One advantage of using WBC6 with mice is that both organisms are genetically tractable, and conditions for *in vitro* encystation of WBC6 are known; *in vitro* encystation is not yet possible for the assemblage B strain GS (15). These limitations and potential differences between models highlight the need for more direct methods to enumerate parasites *in vivo* and quantify *in vivo* parasite physiology and differentiation.

To assess parasite colonization and differentiation dynamics in the host, we developed bioluminescent imaging (BLI) methods allowing us to directly quantify and image temporal and spatial dynamics of *Giardia* colonization using the genetically manipulable assemblage A isolate WBC6 (35). Specifically, we have infected mice and gerbils with WBC6 trophozoites or cysts expressing firefly luciferase (FLuc) under the control of either constitutive or encystation-specific (41–45) promoters. BLI is used extensively in diverse animal models and enables sensitive quantification and real-time reporting of metabolic activity via imaging of the transcriptional activity of promoter-luciferase fusions (46–48). Protein expression can also be monitored (49). BLI has been used previously to monitor *in vivo* parasite metabolism and infection dynamics in animal models of malaria, leishmaniasis, trypanosomiasis, and toxoplasmosis (50–52), as well as bacterial colonization of the intestine (41).

Using noninvasive imaging of bioluminescent *Giardia* parasites, we show real-time parasite physiology in the host, allowing us to confirm and extend early observations of *G. muris* or *G. lamblia* colonization of the proximal small intestine of mice (53), gerbils (54), and humans (55). We also improve our understanding of the *in vivo Giardia* life cycle, demonstrating that encystation is initiated early in the course of infection, peaks within the first week, and is correlated with the highest parasite density during infection. Contrasting studies have reported that parasites colonize the midjejunum of adult and immunodeficient mice (16) and that parasites encyst in the ileum and colon, due to the identification of cysts in distal anatomical sites of the gastrointestinal tract (19, 53).

Last, we demonstrate that high parasite density contributes to the induction of encystation-specific transcription *in vitro*. Thus, local regions or foci of high parasite density *in vivo* may directly (or indirectly) contribute to the early *in vivo* differentiation of parasites that we observed in mice. In total, we show the utility of BLI to evaluate *in vivo Giardia* physiology and differentiation in two animal hosts, facilitating quantifiable longitudinal and spatial monitoring of infection dynamics. BLI has been used extensively to evaluate drugs in numerous parasitic and bacterial infections (56); thus, we expect that BLI will be equally valuable as an alternative and real-time method to evaluate antigiardial drugs in relevant animal models of giardiasis.

## RESULTS

### Visualizing and quantifying *Giardia* infection dynamics using noninvasive bioluminescent imaging in mice.

**FIG 1:**
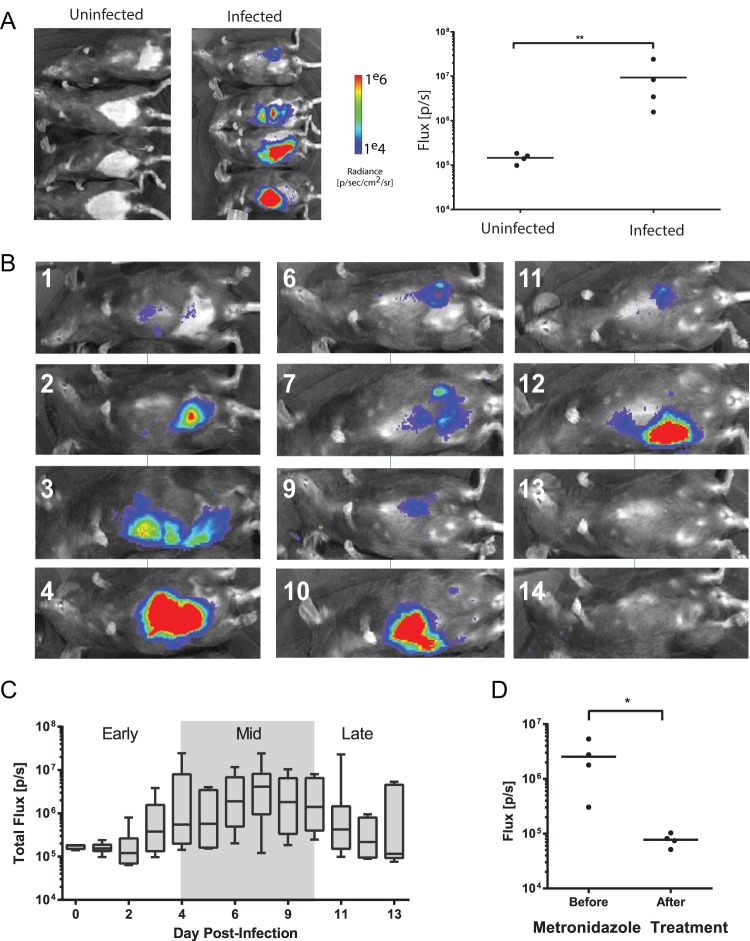
Bioluminescent imaging allows noninvasive quantification of *Giardia* infection dynamics in individual cohorts of mice. (A) A cohort of four mice was inoculated with the *P_GDH_-FLuc* strain, and bioluminescence at 7 days postinoculation was compared to the background signal from uninfected animals (images at left). *P_GDH_-FLuc* bioluminescence intensity is indicated in the image overlay, with the highest signal intensity (radiance, or photons/second/square centimeter/steradian) shown in red and the lowest in blue (see scale bar). Bioluminescence flux (photons/second) rates are compared between the uninfected and infected group, with the asterisks indicating significance as assessed by the ratio unpaired *t* test (*P* < 0.05). (B) Cyclic variability of the *P_GDH_-FLuc* bioluminescence is shown in the same animal imaged noninvasively up to 14 days postinfection. (C) Summary of infection dynamics in two experiments with 8 and 12 mice as quantified by *P_GDH_-FLuc* bioluminescence. The average bioluminescence measured for the cohort each day is shown. The shaded box indicates the maximal bioluminescent signal, or peak infection range. The average time to maximal bioluminescence after infection with the *P_GDH_-FLuc* strain was 6.6 days. (D) A cohort of four mice was imaged, and flux (photons/second) was quantified 5 days after infection with the *P_GDH_-FLuc* strain (Before). The same four mice were imaged, and flux was quantified 2 days after treatment with 50 mg/kg metronidazole by oral gavage (After). The asterisk indicates significance (*P* < 0.05) using an unpaired *t* test.

To confirm that the promoter-firefly luciferase (FLuc) fusions (see Fig. S1 in the supplemental material) are stably integrated and that the bioluminescence is not lost in the absence of antibiotic selection, we used *in vitro* bioluminescence assays to monitor luciferase activity after the removal of antibiotic selection (Fig. S2B). Both *P_GDH_-FLuc* and *P_CWP1_-FLuc* strains maintained a consistent bioluminescence for at least 3 weeks under normal growth conditions (Fig. S2B). Luciferase catalyzes the production of light in the presence of luciferin substrate. While oxygen is required for light production, the colon has sufficient oxygen for detectable light output (57), and d-luciferin delivered by local intraperitoneal injection is rapidly taken up into the entire gastrointestinal tract within 5 min (58). Because *Giardia* trophozoites proliferate in the low-oxygen gut lumen, we tested d-luciferin delivery both orally (by gavage) and systemically (by intraperitoneal injection) to determine the delivery method that produced the optimal bioluminescent signal for *Giardia* colonization (Fig. S3A). Intraperitoneal injection produced a maximal bioluminescence from the *Giardia* bioreporter luciferase strains within 10 min that was stable for over 30 min after injection (Fig. S3B). Uninfected mice or mice infected with a nonluminescent strain of *Giardia* had negligible background signal (Fig. 1A). Last, *in vitro* bioluminescent signal intensity of the *P_GDH_-FLuc* strain is also directly correlated with parasite density in culture (Fig. S4). We show that luciferase continues to be expressed at significant and similar levels both 3 h and 24 h after transfer into encystation medium (Fig. S4).

10.1128/mSphere.00343-16.1 FIG S1  Plasmid maps of the *P_GDH_-FLuc*, *P_CWP1_-FLuc*, and *P_CWP2_-FLuc* constructs for integration into *Giardia*. The plasmid construct *P*_*GDH*_*-FLuc_*5UAUK (A) contains a firefly luciferase gene (*FLuc*) with transcription driven by the *Giardia* glutamate dehydrogenase (GDH) promoter (located within 200 bp upstream of the GDH start codon). A region of *Giardia* DNA, including portions of the *Giardia* aurora kinase (AUK) and nucleolar protein (NOP5) genes, was included to facilitate integration. The plasmid includes an ampicillin resistance gene (AMP) and a puromycin resistance gene (PAC) for selection in *Giardia*. Also shown are maps for the encystation-specific promoter constructs *P*_*CWP1*_*-FLuc_*5UAUK (B) and *P*_*CWP2*_-*FLuc_*5UAUK (C), where CWP1 or CWP2 promoter regions drive expression of *FLuc*. Download FIG S1, EPS file, 1.5 MB. Copyright © 2017 Barash et al. 2017 Barash et al. This content is distributed under the terms of the Creative Commons Attribution 4.0 International license.

10.1128/mSphere.00343-16.2 FIG S2  Optimization and verification of *in vitro* bioluminescence in the *P_GDH_-FLuc* and *P_CWP1_-FLuc* strains. (A) IVIS Spectrum imaging and quantification of bioluminescence of the *P_GDH_-FLuc* strain grown for 3 weeks after withdrawal of puromycin selection and exposed to two concentrations of luciferin prior to imaging. (B) Bioluminescence is quantified in both the *P_GDH_-FLuc* and *P_CWP1_-FLuc* strains in TYI-S-33 (growth) medium (95) and encystation (encyst) medium (61), following withdrawal of antibiotic selection for up to 21 days. (C) The bioluminescent signal (luminescence/second) of the *P_CWP1_-FLuc* strain was upregulated over 400-fold within 10 h of switching to encystation medium. Download FIG S2, EPS file, 1.6 MB. Copyright © 2017 Barash et al. 2017 Barash et al. This content is distributed under the terms of the Creative Commons Attribution 4.0 International license.

10.1128/mSphere.00343-16.3 FIG S3  Optimization and verification of bioluminescence using noninvasive *in vivo* optical imaging of the *P_GDH_-FLuc* and *P_CWP1_-FLuc* strains. Intraperitoneal (i.p.) injection of luciferin substantially increased the bioluminescent signal in a cohort of mice infected with the *P_GDH_-FLuc* strain, compared to oral delivery. (A) Quantification of bioluminescence in two representative animals. (B) The luciferase bioluminescence (photons/second) in two mice infected with the *P_GDH_-FLuc* strain persists for 10 to over 30 min after intraperitoneal injection of luciferin. Download FIG S3, EPS file, 1.5 MB. Copyright © 2017 Barash et al. 2017 Barash et al. This content is distributed under the terms of the Creative Commons Attribution 4.0 International license.

10.1128/mSphere.00343-16.4 FIG S4  The bioluminescent signal intensity of the *P_GDH_-FLuc* strain is correlated with parasite density *in vitro* and is consistent in encystation medium. Luminescence from the *P_GDH_-FLuc* strain was quantified up to 24 h after shifting into encystation medium using two cultures of trophozoites at low (10,000) and high (1,000,000) density. Download FIG S4, EPS file, 1.4 MB. Copyright © 2017 Barash et al. 2017 Barash et al. This content is distributed under the terms of the Creative Commons Attribution 4.0 International license.

To query the temporal sequence of *in vivo* colonization, we infected a cohort of mice with trophozoites of a constitutive bioreporter strain (*P_GDH_-FLuc*) and quantified the bioluminescent signal over a 14-day time course (Fig. 1). At day 7, we observed significant bioluminescence compared to uninfected animals (ratio paired *t* test, *P* < 0.0067). Individual mice showed variation in the degree of bioluminescent signal (Fig. 1A), and some animals exhibited signal periodicity; one representative individual (Fig. 1B) showed bioluminescence peaks at day 4, day 10, and day 12 postinfection (p.i.). Maximum bioluminescence occurred between day 4 and day 9 for all animals infected with the *P_GDH_-FLuc* strain (*n* = 20 over two experiments) (Fig. 1C). To ensure that the bioluminescent signal was attributable to metabolically active parasites, we also treated mice infected with the *P_GDH_-FLuc* strain with 50 mg of metronidazole/kg of body weight by oral gavage. After 2 days of treatment, the bioluminescent signal had decreased to the same level as that of noninfected animals (Fig. 1D).

### Quantifying the spatial variation of *Giardia* infection using *ex vivo* imaging of the murine gastrointestinal tract.

**FIG 2:**
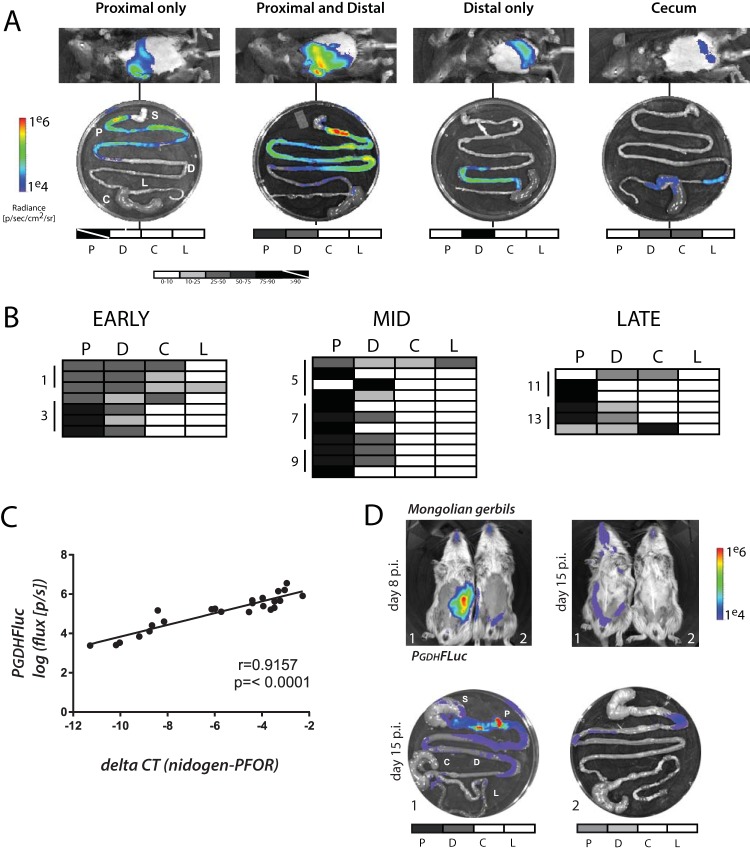
High-density foci of *Giardia* colonization are present in the proximal small intestine of mice and gerbils. (A) Representative classes of *in vivo* and *ex vivo* bioluminescent images are shown for 24 mice infected with the *P_GDH_-FLuc* strain and sacrificed in cohorts of four on days 3, 5, 7, 9, 11, and 13 postinfection. Photon flux or radiance (photons/second/square centimeter/steradian) for each intestinal segment is shown and has been normalized to the maximal *ex vivo* bioluminescence signal on the radiance scale, yielding the percent total signal per segment. These values are represented graphically on the grayscale maps below each *ex vivo* image (white, 0 to 10%; black, 75 to 100%; values between 10% and 75% indicated as shades of gray). A strikethrough indicates greater than 90% of maximal bioluminescent signal. P, proximal small intestine; D, distal small intestine; C, cecum; L, large intestine. The stomach (S) is shown for orientation but always lacks bioluminescence. (B) Quantitative bioluminescence imaging from infections is categorized and summarized by the region of the gastrointestinal tract for all animals in each phase of infection as early (days 0 to 3), middle (days 5 to 9), and late (days 11 to 13). Shading in each row of the charts indicates the variations in the maximal bioluminescence in each of the four regions in an individual animal. (C) Degree to which the *in vivo P_GDH_-FLuc* bioluminescence is significantly and linearly correlated in 24 intestinal samples from four infected mice with quantitative PCR measures of *Giardia* abundance in the same samples using a single-copy *Giardia* gene (PFOR1) (normalized to the mouse single-copy nidogen gene). Statistical significance (*R* = 0.9157, *P* < 0.0001) is noted. (D) The same *P_GDH_-FLuc* strain was used to infect two Mongolian gerbils (see Materials and Methods) without antibiotic pretreatment, and animals were noninvasively imaged over 15 days. Day 8 p.i. and day 15 p.i. noninvasive whole-animal imaging and *ex vivo* gastrointestinal tract imaging for day 15 p.i. are shown for both animals (1 and 2) with anatomical annotations as in panels A and B.

To assess spatial infection dynamics and to correlate noninvasive imaging with *ex vivo* imaging of excised intestine, we inoculated 21 mice with 1 million *P_GDH_-FLuc* trophozoites. On days 1, 3, 5, 7, 9, 11, and 13 postinfection, three or four animals were individually imaged. Animals were then sacrificed, and the gastrointestinal tracts were quickly excised and imaged* ex vivo* (Fig. 2). We observed four major patterns of bioluminescence within the gastrointestinal tracts over the course of infection (representative patterns are shown in Fig. 2A). The majority of bioluminescent signal occurred in the proximal small intestine as early as 1 day following oral gavage (Fig. 2B), yet there was some spatial variability in the gastrointestinal parasite colonization pattern in the cohorts over the 13 days. Further, we observed localized areas of maximal bioluminescent signal, or foci, within colonized regions of the gut (Fig. 2A). These regions are upward of 100-fold more bioluminescent than adjacent regions in the same anatomical section. In some animals, bioluminescence was present in the distal small intestine or diffuse throughout the small intestine. Less commonly observed was bioluminescence occurring primarily in the cecum or the large intestine. For all samples, BLI signal intensities of less than 1% of total maximal signal were seen within the stomach. The *in vivo* imaging signal intensities were directly comparable with the* ex vivo* imaging (Fig. S5).

**FIG 3:**
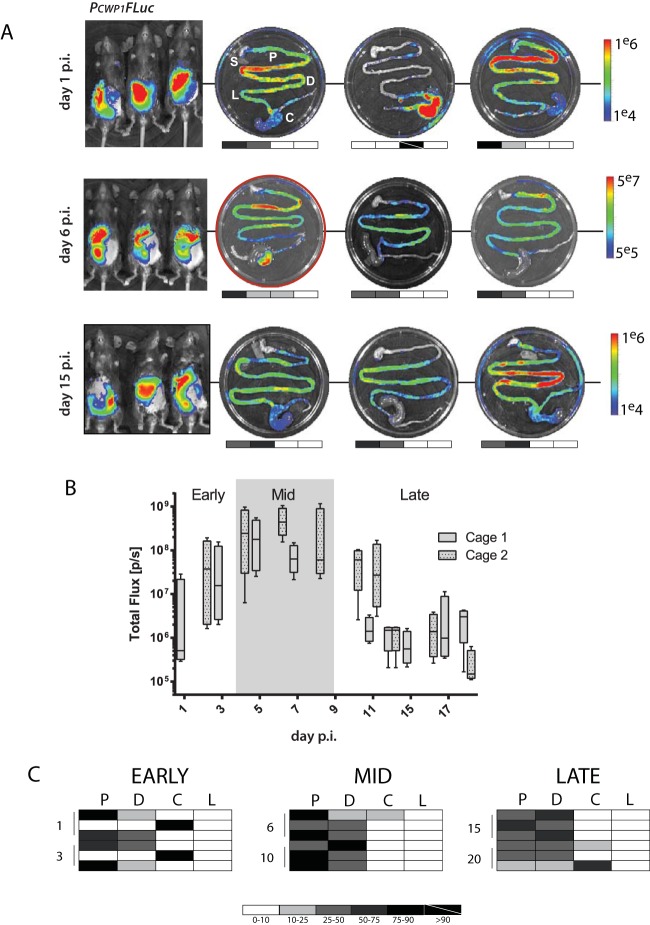
Encystation initiation occurs early in infection in both the proximal and distal small intestine. Eighteen mice were inoculated with the *P_CWP1_-FLuc* strain, and cohorts of three animals were sacrificed and imaged on days 1, 3, 6, 10, 15, and 20 postinfection. (A) The whole-animal* in vivo* images from days 1, 6, and 15, representing early, mid-, and late infection, respectively, are shown with corresponding *ex vivo* images from each animal (S, stomach; P, proximal; D, distal; C, cecum; L, large intestine). The stomach is shown for orientation but always lacks bioluminescence. Days 1 and 15 are presented on a scale between 1e4 and 1e6 photons/s. Day 6 has the maximal signal, and images are presented using a scale between 5e5 and 5e7 photons/s. For each *ex vivo* image, the photon flux (photons/second/square centimeter/steradian) for each intestinal segment is normalized to the maximal *ex vivo* bioluminescence signal on the radiance scale, yielding the percent total signal per segment. Grayscale maps of bioluminescence are shown below each *ex vivo* image (white, 0 to 10%; black, 75 to 100%; values between 10% and 75% are indicated as shades of gray). (B) Two cages of mice (*n* = 4 per cage) were inoculated with the* P_CWP1_-FLuc* strain and imaged every other day. The box-whisker plot summarizes bioluminescent signals for encystation initiation (*P_CWP1_-FLuc*) for each phase of infection (early, days 0 to 3; mid-, days 4 to 9; late, days 10 to 20), with the center line indicating the median total flux (photons/second), and the ends of boxes representing the first and third quartiles below and above the median, respectively. (C) The spatial localization of signal is summarized for each individual animal in each row. The shaded charts summarize the percentage of maximal bioluminescent signal from the *P_CWP1_-FLuc* strain in each of the four gastrointestinal regions (P, proximal small intestine; D, distal small intestine; C, cecum; L, large intestine) for all infected animals in early, middle, and late stages of infection.

10.1128/mSphere.00343-16.5 FIG S5  The bioluminescent signal intensity of the *P_GDH_-FLuc* strain imaged noninvasively *in vivo* is directly correlated with the signal obtained with *ex vivo* imaging. For 20 days, the overall levels of *in vivo* bioluminescence (photons/second) are compared with overall total *ex vivo* bioluminescence of intestinal segments in a cohort of the 18 mice infected with the *P_CWP1_-FLuc* strain (Fig. 3). Representative images compare the *in vivo* imaging bioluminescent signal to that from *ex vivo* imaging of the entire gastrointestinal tract from the same animal. Download FIG S5, EPS file, 1.8 MB. Copyright © 2017 Barash et al. 2017 Barash et al. This content is distributed under the terms of the Creative Commons Attribution 4.0 International license.

Through a comparison of colonization patterns during early, mid-, and late infections (Fig. 2B), we found that early in infection, there was more diffuse small intestinal colonization, with 48% of the BLI signal from all animals localized to the proximal small intestine and nearly one-third of signal from the distal small intestine. At maximal infection (Fig. 1C), the proximal small intestine was more strongly colonized than the distal, accounting for 71% of overall signal. Four of 11 mice (36%) had a proximal-only colonization pattern, with an average of 89% proximal signal among the individuals. Only one mouse had significant colonization of the distal intestine. Late in infection, higher BLI signal intensity was detected in the distal small intestine and cecum, although the proximal small intestine still accounted for 57% of overall signal. Early and late infections were characterized by a more diffuse pattern throughout the gastrointestinal tract, whereas during the maximal infection (midinfection), more parasites were concentrated in the proximal small intestine.

We next interrogated the degree to which the *in vivo* bioluminescence of the *P_GDH_-FLuc* constitutive bioreporter correlated with parasite abundance using quantitative PCR (qPCR) of a single-copy *Giardia* gene (Fig. 2C). We determined that there is a significant and linear association between bioluminescence intensity and infection density as imaged using the *P_GDH_-FLuc* strain (Fig. 2C, *P* < 0.0001). Specifically, following *ex vivo* imaging and quantification of bioluminescence, we quantified total parasites using qPCR of genomic DNA isolated from 24 1-cm intestinal segments in regions of high and low bioluminescent signal in four infected animals (Fig. 2C). We amplified the *Giardia* pyruvate ferredoxin oxidoreductase gene (*PFOR1*) and used the constitutively expressed murine nidogen-1 gene as an internal control to determine the contribution of murine DNA to total genomic DNA isolated from intestinal segments. A smaller difference in differential counts to threshold (Δ*C*_*T*_) between nidogen and *PFOR* genes indicated greater numbers of parasites, as more murine DNA was present than *Giardia* DNA, while a larger difference in Δ*C*_*T*_ indicated fewer parasites.

### Visualizing and quantifying *Giardia* temporal and spatial infection dynamics in Mongolian gerbils.

Four gerbils infected with *P_GDH_-FLuc* exhibited bioluminescence and were imaged both noninvasively *in vivo* and terminally using *ex vivo* imaging of isolated gastrointestinal tracts (Fig. 2D). Infected gerbils were strongly bioluminescent after 8 days of infection, and infections had decreased by 15 days postinoculation. As in mice, *ex vivo* imaging confirmed that* Giardia* primarily colonized the proximal small intestine in high-density foci, as seen with localized regions of bioluminescence.

### Encystation occurs early in infection in both the proximal and the distal small intestine in mice.

*Giardia* cysts consist of a partially divided trophozoite surrounded by a desiccation-resistant cyst wall that is composed predominantly of leucine-rich cyst wall proteins (CWPs). CWPs are transported to the outer membrane by encystation-specific vesicles (ESVs) approximately 2 to 3 h after transfer to *in vitro* encystation medium (24, 59). Cyst wall protein 1 (CWP1) expression is upregulated over 100-fold within 7 h after switching to *in vitro* encystation medium (60, 61). The bioluminescent signal from the *P_CWP1_-FLuc* strain increased 400-fold when transferred to *in vitro* encystation medium (Fig. S2C). We also show that *P_CWP1_-FLuc* retains the ability to upregulate expression from the CWP1 promoter after shifting the strain to encystation medium following 3 weeks of serial passage of this strain without antibiotic selection in nonencystation medium (Fig. S2B).

To determine the temporal and spatial dynamics of *Giardia* encystation *in vivo*, we inoculated eight mice with 1 million *P*_*CWP1*_*-FLuc*-expressing trophozoites. *P_CWP1_-FLuc* bioluminescence was quantified every other day in live animals. One day postinfection, we observed significant *P_CWP1_-FLuc* signal (Fig. 3A), comparable to *in vitro* transcriptional upregulation of CWP1 (Fig. S2) (24, 61). The maximal bioluminescence from the *P_CWP1_-FLuc* bioreporter occurred at 6 days postinfection, and significantly high bioluminescence ranged from 5 to 8 days postinoculation (Fig. 3B). While the *P_CWP1_-FLuc* bioluminescence from all animals was highest within the first week of infection, the bioluminescence was detectable throughout the 17 days of infection, including day 1 (early infection), day 6 (midinfection), and day 15 (late infection) (Fig. 3).

To determine the regions of the murine gut where encystation is initiated, cohorts of three animals were sacrificed on days 1, 3, 6, 10, 15, 20, and 26 postinoculation with the encystation bioreporter strain *P_CWP1_-FLuc*, and the entire gastrointestinal (GI) tract was imaged and scored by region (Fig. 3A). Upregulation of the *P_CWP1_-FLuc* encystation bioreporter was detectable* ex vivo* as early as day 1 postinfection. Maximal *P_CWP1_-FLuc* bioluminescence was primarily observed in the proximal small intestine, 3 to 5 cm distal to the stomach, as observed for the constitutive *P_GDH_-FLuc* bioreporter strain. Like *P_GDH_-FLuc*, *P_CWP1_-FLuc* bioluminescence was often observed as regions of local maxima or foci within an area of lower bioluminescence (Fig. 2 and 3).

In contrast to the *P_GDH_-FLuc* bioreporter strain, bioluminescence from the encystation bioreporter *P_CWP1_-FLuc* was more distributed throughout the small intestine (Fig. 3A). Early in infection, equal numbers of mice displayed *P_CWP1_-FLuc* bioluminescence in the proximal and distal small intestines (SIs) (Fig. 3C), and yet the bioluminescence from *P_CWP1_-FLuc* localizing to the proximal SI accounted for 50% of total intensity, whereas the distal SI signal was only 16% of the total bioluminescence in the gut. At mid- and late infection, proximal and distal SI signal intensities were comparable (54% and 40%, peak; 37% and 44%, late, respectively) with equal numbers of mice showing signal from both proximal and distal SI regions.

We also noted increased localization of *P_CWP1_-FLuc* bioluminescent signal to the cecum, compared to *P_GDH_-FLuc*, which localized primarily to the proximal small intestine (Fig. 2). Two mice from early infection and one from late infection had strong cecal bioluminescent signals, sometimes at the exclusion of other anatomical sites, or in conjunction with bioluminescence elsewhere in the gastrointestinal tract.

### Confirmation of encystation initiation in the proximal small intestine during early infection in mice.

To confirm the encystation initiation pattern early in infection, we infected animals with a second encystation-specific strain, *P_CWP2_-FLuc*, containing the promoter region of the cyst wall protein 2 (CWP2) gene (31). The temporal and spatial dynamics of encystation initiation that we observed with *P_CWP2_-FLuc* were similar to those of *P_CWP1_-FLuc* (Fig. S6).

10.1128/mSphere.00343-16.6 FIG S6  Bioluminescent imaging of the *P_CWP2_-FLuc* strain confirms that encystation initiation occurs early in infection in both the proximal and distal small intestine. Mice were inoculated with the encystation-specific *P_CWP2_-FLuc* strain, and cohorts of three animals were sacrificed and imaged on days 1, 4, and 7 postinfection. (A) The box plot summarizes bioluminescent signal for encystation initiation (*P_CWP2_-FLuc*) for the first 7 days of infection. (B) The whole-animal* in vivo* images from days 1, 4, and 7, representing early infection and midinfection, are shown with the corresponding *ex vivo* images from day 7 p.i. for each animal (S, stomach; P, proximal; D, distal; C, cecum; L, large intestine). The quantitative BLI signal is categorized by the region of the gastrointestinal tract, and the overall spatial localization of signal is summarized for each individual animal. Grayscale heat maps of bioluminescence are shown below each *ex vivo* image (white, 0 to 10%; black, 75 to 100%, with values between 10% and 75% indicated as shades of gray). (C) *In vitro* bioluminescence assays compare the relative signal intensities of various cell densities of the* P_CWP1_-FLuc* strain compared to the *P_CWP2_-FLuc* strain. Download FIG S6, TIF file, 1.9 MB. Copyright © 2017 Barash et al. 2017 Barash et al. This content is distributed under the terms of the Creative Commons Attribution 4.0 International license.

**FIG 4:**
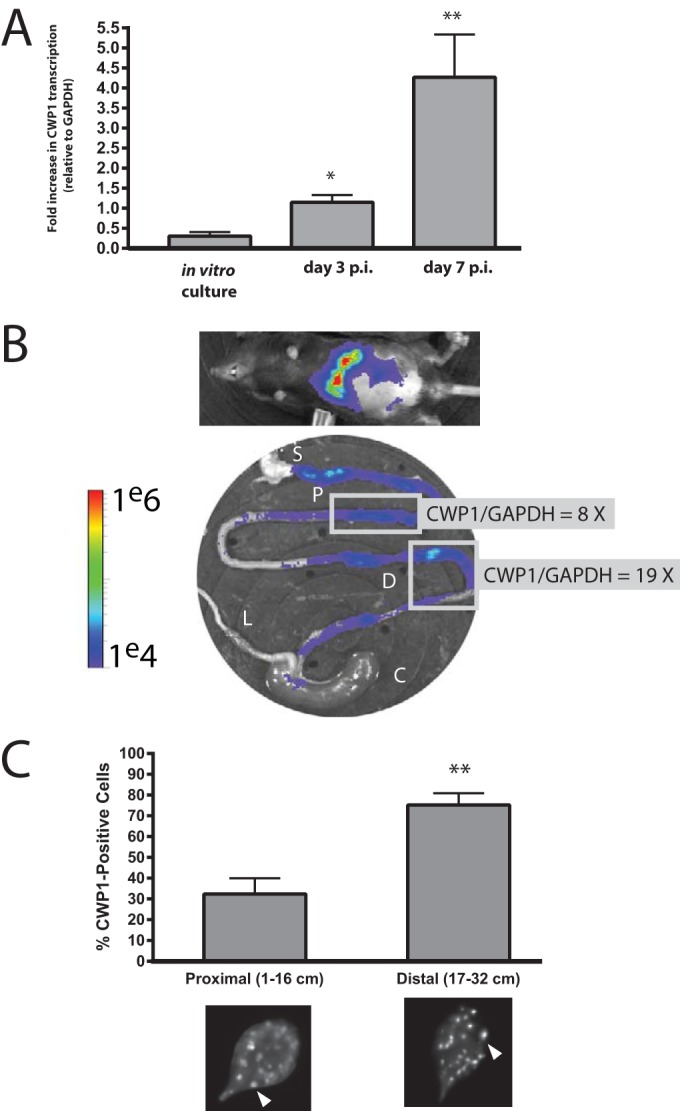
CWP1 qPCR and immunostaining of ESVs verify encystation in the proximal and distal small intestine. (A) Quantitative PCR was used to compare the *in vivo* expression of CWP1 relative to GAPDH (CWP1/GAPDH) in the first 5 cm of the small intestine at days 3 and 7 p.i. to that of a confluent *in vitro* culture. (B) A representative animal infected with the *P_CWP1_-FLuc* strain is imaged noninvasively at day 7 p.i., and *ex vivo* BLI of the gastrointestinal tract (P, proximal small intestine; D, distal small intestine; C, cecum; L, large intestine; S, stomach) is shown with corresponding CWP1/GAPDH qPCR of representative proximal and distal small intestine regions (boxed). (C) Encystation-specific vesicles (ESVs) were immunostained using an anti-CWP1 antibody and quantified in pooled *ex vivo* samples from two animals each at days 3 and 7 p.i. ESVs are marked by arrowheads in representative images from each respective intestinal sample. The percentages of trophozoites positive for ESVs are shown as a percentage of total trophozoites imaged (*n* > 600 cells counted) in regions of the proximal small intestine and distal small intestine. Asterisks indicate statistical significance using unpaired *t* tests with Welch’s correction; *, *P* < 0.05; **, *P* < 0.005, compared to *in vitro* culture.

Because infections with both encystation-specific *P*_*CWP1*_-*FLuc* and *P_CWP2_-FLuc* bioreporter strains indicated that encystation initiation occurs early during infection and is primarily localized to the proximal SI, we confirmed the expression of CWP1 transcripts throughout the gut using qPCR of *ex vivo* samples following bioluminescent imaging. Within the first 5 cm of the proximal SI, transcription of CWP1 was upregulated by 3 days postinfection, with significantly more upregulation by day 7 relative to basal CWP1 transcription levels in *in vitro* culture (Fig. 4A).

Upregulation of encystation-specific promoter activity results in the commitment of trophozoites to differentiate into cysts that are shed into the environment to infect new hosts (61). Hallmarks of this commitment to encystation include the upregulation of CWP1 and CWP2 genes and the appearance of encystation-specific vesicles (ESVs) that transport the cyst wall proteins (e.g., CWP1 and CWP2) to build the cyst wall (62, 63). We find that *in vivo* CWP1 gene expression corresponds to the *in vivo* BLI signal of the *P_CWP1_-FLuc* strain. As previously shown in Fig. 3, the *P_CWP1_-FLuc* bioluminescence is localized in foci throughout in the proximal and distal small intestine (Fig. 4B). We observed a significant increase in CWP1 gene expression (relative to glyceraldehyde-3-phosphate dehydrogenase [GAPDH]) in these foci of the distal small intestinal and proximal small intestinal regions. Specifically, we quantified 8-fold- and 19-fold-higher CWP1 gene expression in the proximal and distal small intestine, respectively, relative to GAPDH expression.

During* in vitro* encystation, ESVs appear within several hours following transfer to encystation medium (18, 21, 61). On days 3 and 7 postinfection, we immunostained the contents of *ex vivo* intestinal samples using an anti-CWP1 antibody (63) and confirmed that trophozoites with encystation-specific vesicles (ESVs) were also present throughout the small intestine (Fig. 4C). CWP1-positive cells represented approximately 80% of the total cells imaged in the distal small intestine (Fig. 4C). Specifically, each trophozoite examined from the small intestine had over 20 ESVs per cell (representative images in Fig. 4C).

### Infection dynamics are similar when trophozoite inoculum size is varied and when infection is initiated with cysts.

**FIG 5:**
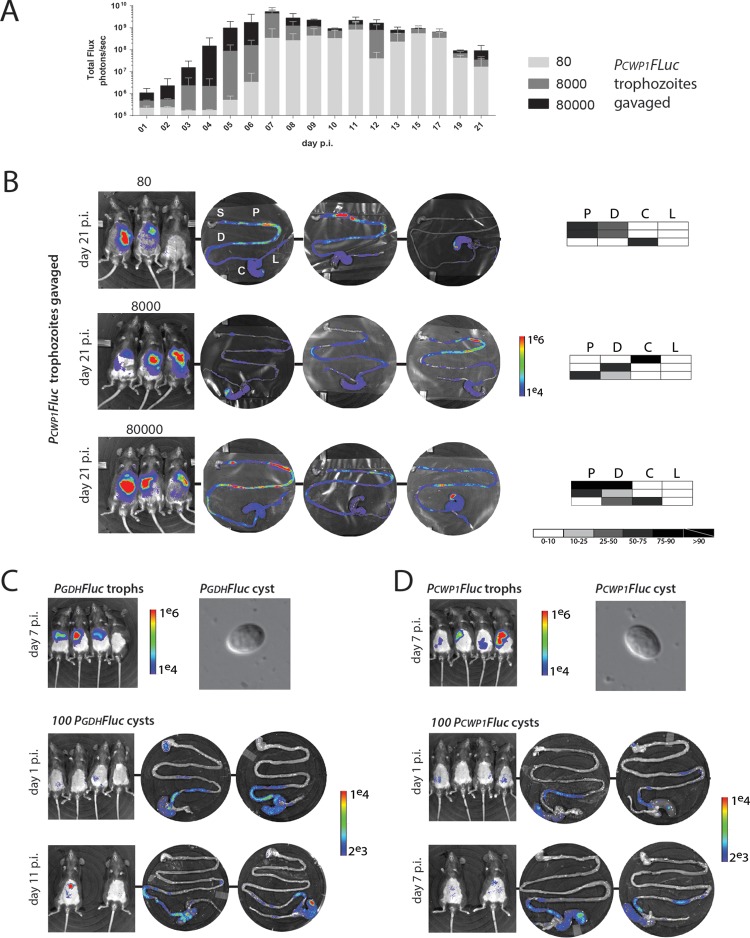
Variations in trophozoite inoculation density or infections with cysts result in similar temporal and spatial dynamics. (A) To assess the impact of cell density on the initiation of encystation, cohorts of three mice were infected with three different concentrations of *P_CWP1_-FLuc* strain trophozoites (80, 8,000, or 80,000), and bioluminescence was imaged and quantified daily over 21 days in total. (B) *In vivo* and *ex vivo* BLI is presented following sacrifice at day 21 p.i. for each inoculation density. The shaded charts summarize the percentage of maximal bioluminescent signal from the *P_CWP1_-FLuc* strain in each of the four gastrointestinal regions (P, proximal small intestine; D, distal small intestine; C, cecum; L, large intestine) for each individual animal infected with that initial inoculum. The stomach (S) is shown for orientation but always lacks bioluminescence. (C) A cohort of mice was infected with the *P_GDH_-FLuc* strain. Cysts were harvested throughout the infection from feces (see Materials and Methods), and 100 cysts were used to infect an additional cohort. Noninvasive imaging of infections using whole-animal BLI and *ex vivo* imaging of the gastrointestinal tract are shown for days 1 and 11 p.i. (D) A similar study was performed using the encystation-specific *P_CWP1_-FLuc* strain (days 1 and 7 p.i. shown).

*Ex vivo* spatial imaging of bioluminescence showed that trophozoite colonization of the host gut is not uniform; rather, vegetative and encysting trophozoites are concentrated in foci, primarily within the proximal small intestine (Fig. 2, 3, and S6). Localized areas of increased parasite density might affect the physiology or differentiation of parasites in this particular region, perhaps contributing to developmental transitions. To assess whether the observed encystation promoter activity in mice was a consequence of initial concentrations of trophozoites used during gavage, we inoculated cohorts of mice (*n* = 4 mice per group, *n* = 12 total) with three different densities of *P_CWP1_-FLuc* trophozoites (Fig. 5A). *P_CWP1_-FLuc* signal intensity was dependent on inoculum density during the first 6 days postinoculation. After day 6, the bioluminescent signal reached maxima that were similar for all three inoculum densities, with a slight and gradual decline over the next 2 weeks (Fig. 5A). Of eight mice imaged daily for 14 days, the maximum bioluminescence was reached at an average of 6 days, with a range between 5 and 8 days. At day 21 postinfection, regardless of initial inoculation density, the *ex vivo* bioluminescent signal primarily remained in the proximal and distal small intestine (Fig. 5B), although some animals had distal or cecum bioluminescence. We suggest that once the initial inoculum reaches a colonization density threshold, perhaps localized to foci, encystation initiation occurs at the maximal level.

*Giardia* infections are routinely initiated by ingesting cysts. We isolated* P_GDH_-FLuc* or *P_CWP1_-FLuc* cysts from feces of mice in order to evaluate infection dynamics and the use of BLI when infecting with a low number of cysts (100 cysts/mouse). Similarly to infection with trophozoites, we observed areas of local signal maxima throughout the gastrointestinal tract for both strains (Fig. 5C and D). *Giardia* colonization after infection with cysts tended to be more distal, and parasites colonized the cecum in each case (Fig. 5C). Encystation-specific signal (*P_CWP1_-FLuc*, Fig. 5D) was observed as early as 1 day postinfection.

### Increased parasite density contributes to encystation initiation.

**FIG 6:**
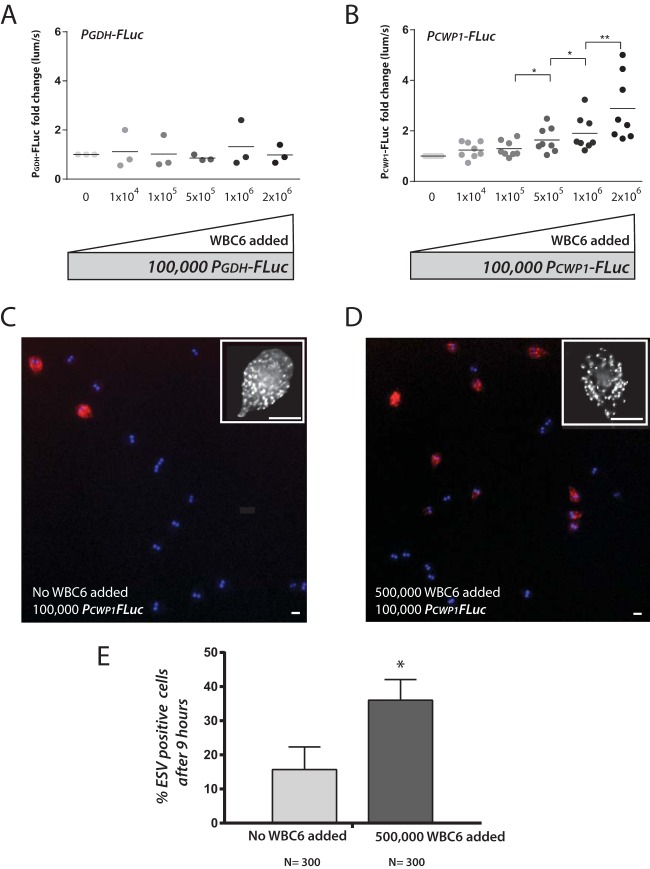
Increased cell density contributes to encystation initiation and upregulation of CWP1 expression. (A and B) Bioluminescence is quantified from an experiment in which 100,000 bioluminescent *P_GDH_-FLuc* (A) or *P_CWP1_-FLuc* (B) trophozoites were incubated up to 12 h in encystation medium, and each well was crowded with increasing numbers (darker-shaded dots) of nonbioluminescent WBC6 parasites (see Materials and Methods). The 9-h time point is shown for both strains; additional time points are presented in Fig. S7 in the supplemental material. *, *P* < 0.05; **, *P* < 0.01. (C and D) Representative images are presented showing the quantification of ESV-positive cells with no nonluminescent WBC6 (wild-type) trophozoites added to 100,000 *P_CWP_-FLuc* strain trophozoites (C) or when 500,000 WBC6 (wild-type) trophozoites were added to 100,000 *P_CWP_-FLuc* strain trophozoites (D). Insets show representative images of ESVs (>100 per cell on average) stained with an anti-CWP1 antibody. Bars, 5 µm. (E) Percentages of ESV-positive cells after 9 h of incubation in encystation medium are compared for the two conditions (C and D). The asterisk indicates significant differences between the two conditions as assessed by the ratio unpaired *t* test (*P* < 0.05).

To evaluate whether parasite density had an effect on the initiation of encystation *in vitro*, we “crowded” cultures of the constitutive bioreporter (*P_GDH_-FLuc*) strain (Fig. 6A) or the encystation-specific bioreporter (*P_CWP1_-FLuc*) strain (9 h, Fig. 6B) with increasing amounts of nonluminescent wild-type WBC6 in encystation buffer (Fig. S7). We then quantified bioluminescence at 3, 6, 9, and 12 h after transfer to encystation medium (9 h [Fig. 6] and 3 to 12 h [Fig. S7]). Within 9 h, we observed a significant increase in bioluminescence from the *P_CWP1_-FLuc* strain with the addition of 5 × 10^5^ to 2 × 10^6^ additional nonluminescent WBC6 trophozoites to the *P_CWP1_-FLuc* strain (Fig. 6B). We observed no increase in bioluminescence with crowding of the *P_GDH_-FLuc* strain (Fig. 6A).

10.1128/mSphere.00343-16.7 FIG S7  Crowding of the *P_CWP1_-FLuc* strain in encystation medium induces *FLuc* expression at higher cell densities. *P_CWP1_-FLuc* trophozoites were incubated up to 12 h in encystation medium, and each well was crowded with increasing amounts (darker-shaded dots) of nonbioluminescent WBC6 parasites. Bioluminescence in wells was quantified at 3, 6, 9, and 12 h. Download FIG S7, EPS file, 1.5 MB. Copyright © 2017 Barash et al. 2017 Barash et al. This content is distributed under the terms of the Creative Commons Attribution 4.0 International license.

To verify that the observed density-dependent increase in *P_CWP1_-FLuc* signal results in a higher proportion of encysting cells, we quantified the proportion of ESV-positive cells in wells containing only luminescent cells (100,000 *P_CWP1_-FLuc* cells) compared to more crowded wells (100,000 *P_CWP1_-FLuc* cells with 500,000 WBC6 cells) after 9 h in encystation medium (Fig. 6C and D). Crowded wells contained a significantly higher proportion of ESV-positive cells than less crowded wells (Fig. 6E).

## DISCUSSION

Limitations and potential differences between different animal models of giardiasis underscore the need to quantify *in vivo Giardia* physiology and differentiation beyond just enumeration of trophozoites and cysts. Parasite burden in mice has been most commonly quantified by directly counting trophozoites or, more recently, by *Giardia*-specific qPCR of intestinal segments (3, 64). In live animals, quantification of fecal cysts is commonly used to estimate parasite abundance, yet cyst shedding is not necessarily a proxy for overall parasite burden or metabolic activity (65).

Overall, bioluminescence imaging of *Giardia* infection provides a real-time, temporal and spatial interrogation of parasite metabolic activity and differentiation (Fig. 1, 2, and 3). We have shown that bioluminescence imaging of an integrated luciferase reporter construct driven by the native glutamate dehydrogenase (GDH) promoter directly correlated with *in vivo* parasite density in mice (Fig. 2C). Importantly, we show that luciferase expression from the GDH promoter continues at significant levels for at least 24 h after the *P_GDH_-FLuc* strain is transferred to encystation medium (see Fig. S4 in the supplemental material). Thus, BLI of constitutive metabolic genes could also be used as a proxy for *Giardia* abundance when properly calibrated to other methods of parasite enumeration.

Using the constitutively expressed *P_GDH_-FLuc* strain, we confirmed maximal infection at approximately 7 days, consistent with prior studies of giardiasis in mice (36). This live-imaging strategy provides the first real-time visualization of the spatial and temporal dynamics of giardiasis *in vivo*, allowing us to assess the timing and location of parasite differentiation. Noninvasive *in vivo* BLI relies on the external detection of light produced internally, and signal intensity may be limited by the overall level of luciferase expression, the oxygen tension within relevant tissues, pigmentation of organs and skin, or any background signal from the animal (42). However, the gut is sufficiently oxygenated to permit signal detection, and while animal tissues exhibit relatively high background levels of autofluorescence, they have nearly nonexistent levels of autoluminescence, which facilitates detection even at low signal strength (42, 44, 66).

*Giardia* cysts are shed sporadically and sometimes cyclically (67, 68). As reported in human giardiasis (69), we observed variability in infections between isogenic cage mates, including variations in the time to maximum infection and spatial colonization patterns, and cyclical infections (Fig. 1 to 3). We confirmed that early infection dynamics vary based on the number of trophozoites inoculated or if cysts are used to initiate the infection, but that as a whole and as previously reported, parasite burden peaks at about 1 week after infection and is most commonly associated with the proximal and distal small intestine (Fig. 5).

### Reassessing the spatial dynamics of *Giardia* physiology and differentiation in two animal hosts.

The convoluted route of the animal gut and the diffusion and refraction of bioluminescence present a challenge when imaging *Giardia* infections in live animals (41). Differentiating localized parasite activity from diffuse infection using *in vivo* optical imaging is also challenging (Fig. 1 and 2).

*Giardia* colonizes the gastrointestinal tract of both mice and gerbils with a localized or “patchy” distribution, as has been observed for many other pathogens of the gastrointestinal tract or other organs (50–52, 66, 70). We show that *Giardia*, rather than uniformly colonizing throughout a region of the GI tract, colonizes the intestine in discrete foci (Fig. 2). Based on early studies using direct counting of trophozoites from intestinal samples, *Giardia* has generally been assumed to primarily colonize the midjejunum, or middle section of the small intestine (16, 53, 71), although other early work suggested that trophozoites prefer to colonize throughout the proximal small intestine (16). We suggest that BLI-directed *ex vivo* sampling of high-density *Giardia* foci could improve the accuracy and sensitivity of subsequent histological or physiological analyses.

Encystation initiation also occurs in discrete foci within the proximal and distal small intestine, or occasionally the cecum (Fig. 3 and S6). Using *ex vivo* BLI, we imaged discrete foci of encysting trophozoites in the proximal small intestine and less commonly in the cecum, and foci were never present in the large intestine. These observations challenge conventional assumptions that chemical cues in the distal gut are solely responsible for the initiation of *in vivo* trophozoite differentiation to the cyst in the *Giardia* life cycle. We found that metabolically active trophozoites are predominantly located in the proximal small intestine, with areas of local intensity frequently just distal to the pylorus (Fig. 2 and 3). We also observed spatial variability between individuals, from diffuse infection throughout the small intestine to patchy foci only in the distal small intestine or cecum. Maximal bioluminescence (and thus infection) correlated strongly with proximal small intestinal colonization, whereas developing or clearing infections were present more diffusely throughout the gastrointestinal tract (Fig. 2 and 3).

The Mongolian gerbil or jird (*Meriones unguiculatus*) is a less commonly used, yet promising animal model for giardiasis (72). Gerbils are often used in the study of both bacterial infections (73, 74) and parasitic infections (75, 76). Gerbils are readily infected by both assemblage A (WBC6) and assemblage B (GS) strains, though infection clearance is delayed with GS (40). Gerbils infected with *Giardia* have been noted to exhibit comparable infection time courses regardless of inoculation stage (cysts versus trophozoites) or site (intragastric versus duodenal) (72). Most notably, in contrast to mice, gerbils do not require antibiotic pretreatment to develop robust infections with the WBC6 strain and also have symptoms of giardiasis consistent with human infection, including wasting, impaired small intestinal disaccharidase activities, and reduced microvillus border surface (77). Here, we show that the constitutive bioreporter *P_GDH_-FLuc* has similar localizations to the proximal small intestine in both mice and gerbils (Fig. 2D), and the reported infection dynamics are similar to that observed in humans (67). Though gerbils provide an excellent model system to study assemblage A or B infections in a natural host, there are no genetic tools available for gerbils. We expect future studies to directly compare infection dynamics in different animal hosts using the same bioreporter strains with subsequent BLI.

### High-density foci of parasites contribute to encystation initiation.

The prevailing view of trophozoite differentiation to cysts in the host has been extrapolated from the chemistry of the gut region where trophozoites were previously believed to encyst (15–17). Parasite commitments to encystation and excystation are key events in *Giardia*’s life cycle, and it is clear that these transitions are highly regulated (61). Premature or tardy encystation can limit cyst development, proliferation, transmission into the environment, and dissemination to new hosts (65). The transition to the cyst form begins with detection of encystation stimuli, resulting in transcriptional upregulation of encystation-specific cyst wall proteins (CWPs) (24, 59). Almost 30 years ago, Gillin et al. showed that elevated bile concentrations could induce encystation in *in vitro* culture (19, 71). When these *in vitro* encystation protocols are used, CWPs are transported to the outer membrane via encystation-specific vesicles (ESVs) within roughly 6 h of exposure to encystation stimuli (24, 59). However, differentiation to cysts can be induced even in the absence of bile, and several *in vitro* culture protocols have been developed to induce encystation by modifying pH, bile, lactic acid, and lipid concentrations in culture (17, 24). Importantly, no *in vitro* encystation protocol produces a high abundance of infectious cysts, suggesting that *in vitro* encystation may not accurately recapitulate differentiation *in vivo*.

Our quantitative *in vivo* imaging of temporal and spatial dynamics of parasite proliferation and encystation implies that there could be other factors contributing to *Giardia*’s developmental transitions during its life cycle in the host. Rather than encystation uniformly occurring throughout a particular region of the gut, we observed nonuniform foci of bioluminescence in infections with CWP1 and CWP2 strains (Fig. 3 and S6). We also detected significant expression of CWP1 in *ex vivo* samples associated with increased bioluminescence in both the proximal and distal small intestine (Fig. 4B). Last, we confirmed the presence of ESVs in trophozoites isolated from the proximal and distal small intestine (Fig. 4), indicating that encystation is initiated and proceeds normally in these regions. In contrast with prior studies, this initiation of encystation occurred early in infections in the proximal small intestine (Fig. 3) and peaked within the same time as maximal parasite density observed using the constitutively expressed *P_GDH_-FLuc* bioreporter strain (Fig. 1). While there was initial variation in the encystation bioluminescent signal proportional to the amount of initial inoculum, we saw that the encystation-specific BLI signal peaked at about 7 days and the encystation BLI signal persisted throughout the 21 days of infection for all inoculum densities (Fig. 5A).

Pathogens have evolved to take advantage of the discrete mucosal surfaces and functions associated with the various anatomical regions of the mammalian gut (78). Reaching a particular threshold of cell density is known to either directly or indirectly modulate developmental programs in diverse parasitic (79–81) and free-living (82) eukaryotes. Density-dependent quorum sensing is key to slender-to-stumpy differentiation in trypanosomes, for example, and trypanosomes can also respond to or affect bacterial quorum sensing signals (80, 81). Parasites such as *Giardia* detect and respond to a variety of chemical and environmental cues during their life cycles, and *Giardia* has been shown to respond to alterations in lipid and pH concentrations *in vitro*, triggering encystation. Alternatively, foci of high parasite density could limit local concentrations of nutrients or metabolites or alter local pH—all of which are reported stimuli for encystation initiation (61). This model of localized parasite density-induced encystation due to the localized depletion of nutrients or accumulation of waste products is congruent with observed *in vitro* contributions of pH and/or lipid starvation to encystation initiation (15–17).

By quantifying both CWP1 expression and the proportion of ESV-positive cells (Fig. 6), we show that high parasite density contributes in part to the initiation of encystation *in vitro*. Encystation thus may be initiated *in vivo* in localized areas of the gut within the discrete high-density regions of *Giardia* colonization (Fig. 3 and 5). We suggest that the nonuniform foci of encystation-specific bioluminescence represent “hot spots” of encystation in the gut. Compared to regions colonized with a lower parasite density, higher-density *Giardia* foci could directly impact the local chemistry of the gut, the commensal microbiome, or the host epithelium (Fig. 2). While we observe some initiation of encystation within the 1st day of infection (Fig. 3), the overall process of encystation in the host is lengthy, and it may take hours before mature, infectious cysts are observed in the large intestine or are recovered in feces. Further characterization of parasite physiology and differentiation in high-density foci compared to low-density regions of colonization will help to elucidate the contribution of parasite density to *Giardia*’s developmental transitions.

### A new tool for evaluating chronic giardiasis and for antigiardial drug screening.

Human giardiasis typically resolves within a few weeks, yet chronic or variable infections can occur (83) and have been linked to impaired physical and cognitive development in children (6). *In vivo* BLI offers both real-time and long-term or longitudinal monitoring of the infection dynamics in mice or gerbils. We monitored and quantified the extent of variation in the *in vivo* expression of *Giardia* metabolic and encystation genes for up to 3 weeks using cohorts of mice infected with one of three *Giardia* bioluminescent reporter strains (*P_GDH_-FLuc*, *P*_*CWP1*_-*FLuc*, or *P_CWP2_-FLuc*). As we have shown, BLI of *Giardia* infection dynamics provides a robust method to estimate variance within such cohorts of study animals. Defining the range and variation of *Giardia* colonization in animals is essential before performing a power analysis to determine the numbers of animals that would be statistically informative. In addition, animal numbers can be reduced with longitudinal BLI of giardiasis—a primary goal of ethical animal use in research (42, 84). We anticipate that the use of dual- or triple-spectrum bioreporter strains (85–87) will permit simultaneous visualization of two or more *Giardia* processes (e.g., metabolic activity and encystation) in the same study animal.

Growing evidence of drug resistance in *Giardia* underscores the need to develop new therapeutic alternatives for the treatment of giardiasis (83), and *in vivo* bioluminescence imaging of murine or gerbil *Giardia* infections will aid in the evaluation of promising antigiardial drug candidates. As we have shown, the BLI of luciferase-expressing strains not only facilitates the monitoring of parasite burden but can also provide real-time information on other aspects of parasite physiology and metabolism. We have validated the use of BLI for the analysis of anti-*Giardia* drugs by demonstrating that metronidazole, the standard-of-care anti-*Giardia* drug that targets parasite metabolic activity (88), reduced *in vivo* bioluminescence of the constitutively expressing *P_GDH_-FLuc* bioreporter strain. Other bioluminescent reporter strains could be utilized for high-throughput *in vitro* screens of candidate drugs, prior to *in vivo* assessment in animal models. BLI studies with anti-*Giardia* drugs targeting nonmetabolic parasitic cellular processes (e.g., motility or encystation) could identify adjunct or complementary treatments that reduce parasite proliferation, infection duration, or cyst dissemination.

## MATERIALS AND METHODS

### Luciferase strain construction and validation.

We created three strains of *Giardia lamblia* WBC6, each with firefly luciferase (FLuc) driven by a specific *Giardia* gene promoter (see Fig. S1 in the supplemental material). FLuc promoter fusion constructs were integrated into the genome as previously described (45). To quantify colonization and metabolic activity, we integrated a construct containing FLuc driven by the constitutive NADP-specific glutamate dehydrogenase (GiardiaDB GL50803_21942) promoter (*P_GDH_-FLuc*) (Fig. S1A). To quantify *in vivo* encystation dynamics, we integrated constructs containing FLuc with the encystation-specific cyst wall protein 1 (GiardiaDB GL50803_5638) promoter (*P_CWP1_-FLuc*) and the encystation-specific cyst wall protein 2 (GiardiaDB GL50803_5435) promoter (*P_CWP2_-FLuc*) (Fig. S1B and C). Briefly, a vector previously used to integrate hemagglutinin (HA)-tagged aurora kinase (89) was modified to contain the coding sequence for firefly luciferase fused to the GDH, CWP1, or CWP2 promoter. Puromycin (Pur^r^) and ampicillin (Amp^r^) resistance cassettes allowed selection in *Giardia* and *Escherichia coli*, respectively. The vector was linearized using MluI, and 10 µg of DNA was electroporated into *Giardia lamblia* strain WBC6 (45). Transfected cells were selected for 7 to 10 days using puromycin (50 µg/ml). Confirmation of successful genomic integration was obtained by PCR amplification (data not shown), as well as *in vitro* bioluminescence assays in vegetative cells (*P_GDH_-FLuc*) and encysting strains (*P_CWP1_-FLuc* and *P_CWP2_-FLuc*) (Fig. S2).

### *Giardia* trophozoite and encystation culture conditions.

*G. lamblia* (ATCC 50803) WBC6 *P_GDH_-FLuc*, *P_CWP1_-FLuc*, and* P_CWP2_-FLuc* strains were cultured in modified TYI-S-33 medium supplemented with bovine bile and 5% adult and 5% fetal bovine serum (56) in sterile 16-ml screw-cap disposable tubes (BD Falcon) and incubated upright at 37°C without shaking. Encystation was induced *in vitro* by decanting TYI-S-33 medium from 24-h cultures (roughly 30% confluent) and replacing it with encystation medium modified by the addition of 0.5 g/liter bovine bile, pH 7.8 (61). After 24 h, cysts settled at the bottom of the tube.

### *Giardia in vitro* bioluminescence and density dependence assay.

To assess the stability of luciferase signal in integrated promoter-FLuc strains without selection, luciferase expression in the *P*_*GDH*_-*FLuc* and *P_CWP1_-FLuc* strains was determined before and after passage of the cells in the absence of antibiotic selection (1:25 dilutions daily for 3 weeks). Confluent tubes were incubated on ice for 15 min to fully detach cells. Cells were pelleted by centrifugation at 900 × *g* for 5 min and resuspended in 1 ml of fresh TYI-S-33 medium supplemented with 150 μg/ml d-luciferin (PerkinElmer). Aliquots (50 µl, three technical replicates) were added to white opaque 96-well microplates (PerkinElmer). Bioluminescence was analyzed on a Victor3 plate reader using 1-s exposures until maximum signal was achieved.

For density dependence assays, wild-type and *P_CWP1_-FLuc* cells were grown to confluence, harvested as described above, and washed and resuspended in encystation medium. One hundred thousand *P_CWP1_-FLuc* cells were plated in each well of a microplate, and a range of dilutions of nonbioluminescent wild-type WBC6 was added to the *P_CWP1_-FLuc* cells in three technical replicates. Encystation medium was then added to adjust the final volume to 200 μl per well. Microplates were individually sealed in type A Bio-Bags (BD) to maintain an anoxic environment and incubated at 37°C for the indicated time points. d-Luciferin was added to 150 µg/ml, and luciferase activity was analyzed as described previously.

### Noninvasive *in vivo* bioluminescent imaging of *Giardia* colonization and encystation in mice and gerbils.

Eight-week-old female C57/B6/J mice (Jackson Laboratory) were maintained on *ad libitum* water and alfalfa-free irradiated rodent pellets (Teklad 2918). To promote parasite colonization, water was supplemented with 1 mg/ml ampicillin and neomycin (Teknova) for 5 days prior to infection (64). Water bottles were kept protected from light to minimize degradation of the antibiotics, and antibiotics were refreshed every other day. Individual mice were marked with ear tags or permanent marker on tails, and hair was removed from the ventral abdomen to facilitate imaging. Each mouse was first shaved using a commercial men’s groomer, and then depilatory cream (Nair) was applied for 30 s. For long-term studies, depilatory cream was reapplied as necessary to maintain a hairless ventral abdomen (41). Each animal was gavaged with 1 × 10^7^ *G. lamblia* trophozoites in 100 μl phosphate-buffered saline as previously described (90). Four- to 6-week-old female Mongolian gerbils (Charles River, Inc.) were maintained as described above except that no antibiotics were supplied to the water. All animal studies were performed with IACUC approval at the University of California, Davis (Scott C. Dawson, Principal Investigator [PI]).

For *in vivo* BLI, animals were sedated using isoflurane (1.5 to 3%) in an induction chamber. d-Luciferin (30 mg/kg of body weight) was then injected intraperitoneally at a dose of 150 mg/kg (total volume injected, 100 μl). Sedated animals were transferred to an optically clear XIC-3 isolation chamber (PerkinElmer) and positioned on their dorsal surface. Bioluminescence was imaged using an IVIS Spectrum (PerkinElmer) with no emission filter. Anesthesia was maintained with 1.5 to 2% isoflurane and 100% oxygen during imaging.

Photons were quantified using an ultrasensitive charge-coupled device (CCD) camera (IVIS Spectrum), and the resulting heat maps of bioluminescent photon emission intensity were overlaid on still images of anesthetized animals. To allow the d-luciferin to distribute throughout the body, images were collected with 2-min exposures constantly over 8 to 10 min until the bioluminescent signal stabilized. The final image collection was performed with 2- to 5-min exposures, dependent on signal strength. Region of interest (ROI) analysis was used to quantify bioluminescence (Living Image). A rectangle encompassing the entire abdomen was drawn for each animal from front paws to anus. BLI data were quantified as total flux (photons/second) for exposure time-independent quantification of signal intensity. For animals infected with *P_GDH_-FLuc*, the minimal signal was normalized to the level of background signal in uninfected mice (1 × 10^4^ photons/s). Because the bioluminescent signal intensity from mice infected with *P_CWP1_-FLuc* was several orders of magnitude stronger than that for mice infected with for *P_GDH_-FLuc*, the minimal threshold signal was adjusted to 5 × 10^5^ photons/s in order to minimize background.

### *Ex vivo* bioluminescence imaging in mice and gerbils.

Sedated animals were euthanized by cervical dislocation. The gastrointestinal tract was quickly dissected from esophagus to anus and positioned within a plastic petri dish. The dish and contents were placed within the XIC-3 isolation chamber, 2.5% oxygen was provided to maximize signal, and the GI tract was imaged with a 30-s exposure. *Ex vivo* imaging was performed less than 30 min after the initial injection of luciferin. ROI analysis was used to quantify bioluminescence (Living Image). Total gastrointestinal tract signal was analyzed with a circle over the entirety of the petri dish. The stomach, proximal SI (first half), distal SI (second half), cecum, and large intestine were traced using the freehand tool.

### *Giardia* cyst collection from murine feces.

Cysts were isolated as previously described (91). Mice were infected with either strain *P*_*GDH*_-*FLuc* or strain *P_CWP1_-FLuc* as described above. Fresh stool was acquired daily by immediate collection after feces exited the animal and was stored at 4°C. After 7 days, a total of 3 g of feces was collected from animals infected with either strain *P*_*GDH*_-*FLuc* or strain *P_CWP1_-FLuc*. Fecal samples were suspended in 10 ml tap water, broken up with a tongue depressor, and filtered through a tea strainer. Fecal solution (5 ml) was layered onto an equal volume of chilled 0.75 M sucrose in a 15-ml Falcon tube. Samples were centrifuged for 5 min at 400 × *g*, and 2 ml of cyst-containing solution was removed from the water-sucrose interface with a sterile transfer pipette. Cysts were quantified visually using a hemacytometer and were diluted to 1,000 cysts/ml with tap water. Cysts were stored at 4°C until use.

### Correlation of *in vivo* parasite density with bioluminescence using qPCR.

One-centimeter segments from a region showing strong *ex vivo* signal were identified, marked in the Living Image software, excised, and flash frozen in liquid nitrogen. Total genomic DNA was extracted using standard methods (92) and diluted to 10 ng/µl in nuclease-free water prior to quantitative PCR (qPCR). Quantitative PCR of the pyruvate-ferredoxin oxidoreductase-1 (*PFOR1*, GiardiaDB GL50803_17063) gene (88) was performed using Pfor1F (5′TTCCTCGAAGATCAAGTTCCGCGT3′) and Pfor1R (5′TGCCCTGGGTGAACTGAAGAGAAT3′) oligonucleotide primers and SensiFAST No-ROX SYBR green master mix in an MJ Opticon thermal cycler, with an initial 2-min denaturation step at 95°C followed by 40 cycles of 95°C for 5 s, 60°C for 10, and 72°C for 10. The single-copy, constitutively expressed murine nidogen-1 (*nid1*) gene was used as an internal control to quantify the contribution of murine DNA to intestinal segments (qPCR primers nidoF [5′CCAGCCACAGAATACCATCC3′] and nidoR [5′GGACATACTCTGCTGCCATC3′]). The differential counts to threshold (Δ*C*_*T*_) between *nid1* and *pfor1* were quantified, and *C*_*T*_ values were determined using the Opticon Monitor software.

### Confirmation of encystation in the proximal small intestine during early infection using qPCR.

A cohort of four mice was infected with *P_CWP1_-FLuc*, and two mice were imaged and sacrificed for sample collection at both day 3 and day 7 postinfection (p.i.). Following *ex vivo* imaging, intestines from infected mice were dissected into 3-cm segments, immediately frozen in liquid nitrogen, and transferred to an −80°C freezer until used for RNA extraction. RNA from intestinal segments was purified using RNA Stat-60 (Tel-Test, Inc.). RNA quality was assessed using spectrophotometric analysis (NanoDrop Technologies) and electrophoresis prior to cDNA synthesis. Double-stranded cDNA was synthesized using the QuantiTect reverse transcription kit (Qiagen). Quantitative PCR of cyst wall protein 1 (CWP1, GiardiaDB GL50803_5638) was performed using cwp1F (5′ TAGGCTGCTTCCCACTTTTGAG 3′) and cwp1R (5′ AGGTGGAGCTCCTTGAGAAATTG 3′) oligonucleotide primers (93) and SensiFAST No-ROX SYBR green master mix in an MJ Opticon thermal cycler, with an initial 2-min denaturation step at 95°C followed by 40 cycles of 95°C for 5 s, 60°C for 10 s, and 72°C for 10 s. The constitutively expressed gene for glyceraldehyde-3-phosphate dehydrogenase (GAPDH; GiardiaDB GL50803_ 6687) was chosen as an internal reference gene and was amplified with gapdh-F (5′ CCCTTCACGGACTGTGAGTA 3′) and gapdh-R (5′ ATCTCCTCGGGCTTCATAGA 3′) oligonucleotide primers. *C*_*T*_ values were determined using the Opticon Monitor software, and statistical analyses were conducted using Prism (GraphPad).

### Immunostaining of encysting trophozoites in intestinal tissue samples and *in vitro* crowding assays.

Intestinal segments from the same infected mice analyzed with qPCR (above) were fixed using 1% paraformaldehyde in 1× HEPES-buffered saline (HBS) as previously described (94). Samples were vortexed to facilitate removal of luminal contents, and supernatant was applied to poly-l-lysine-coated coverslips. Cells were permeabilized in 0.1% Triton X-100 for 10 min. Coverslips were washed three times with 2 ml of PEM buffer (0.1 M PIPES, pH 6.9, 2 mM EGTA, 1 mM MgSO_4_). Immunostaining was performed with a mouse primary antibody to cyst wall protein 1 (Giardi-a-Glo; Waterborne, Inc.) and a donkey anti-mouse secondary antibody conjugated to an Alexa 350 fluorophore (Invitrogen). Cells from crowding experiments were fixed by adding a final concentration of 1% paraformaldehyde directly to plate wells. Cells were washed in PEM buffer, and staining was performed as described above.

Images were acquired via automated MetaMorph image acquisition software (MDS Technologies) using a Leica DMI 6000 wide-field inverted fluorescence microscope with a PlanApo 100×, 1.40-numerical-aperture (NA) oil immersion objective. At least 100 trophozoites were counted per slide, and cells were binned into encysting or normal trophozoite morphologies. Regions of small intestine were distinguished spatially as follows: proximal, 1 to 16 cm; and distal, 17 to 32 cm. Slides from 3 to 6 separate intestinal segments were counted per spatial bin. The statistical significance of differences in cell number between the spatial bins was determined via Student’s *t* test.

### Accession number(s).

Plasmid sequence data have been deposited in GenBank under accession numbers MF062155 to MF062157.
