# Supplementary figures and images for: Giardia Colonizes and Encysts in High-Density Foci in the Murine Small Intestine
Source: mSphere. 2017 Jun 21;2(3):e00343-16. doi: 10.1128/mSphere.00343-16 (PMC5480036; doi:10.1128/mSphere.00343-16)

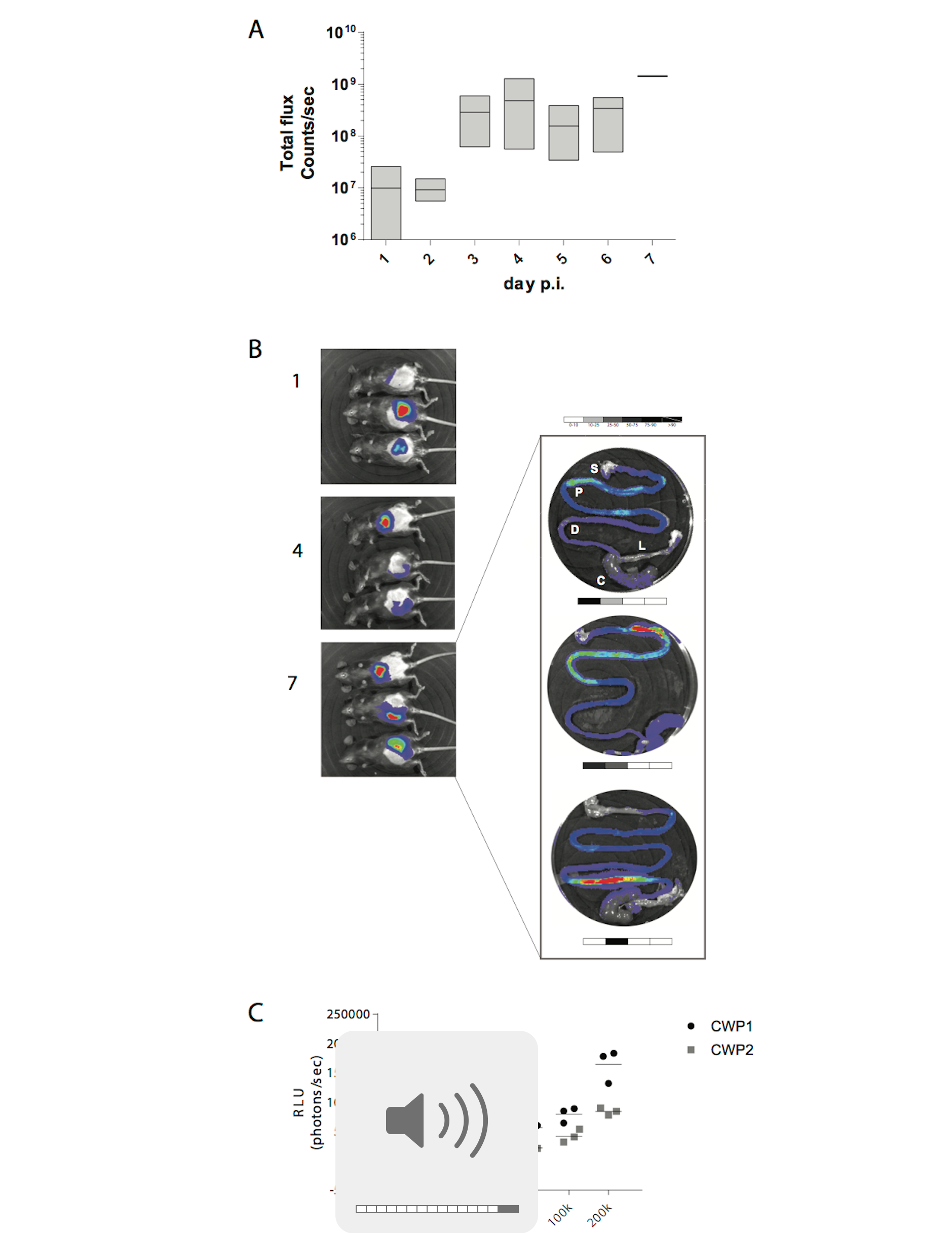

Supplement: FIG S6 [file sph003172287sf6.tif]
